# The androgen-regulated gene human kallikrein 15 (*KLK15*) is an independent and favourable prognostic marker for breast cancer

**DOI:** 10.1038/sj.bjc.6600590

**Published:** 2002-11-12

**Authors:** G M Yousef, A Scorilas, A Magklara, N Memari, R Ponzone, P Sismondi, N Biglia, M Abd Ellatif, E P Diamandis

**Affiliations:** Department of Pathology and Laboratory Medicine, Mount Sinai Hospital, Toronto, Ontario, Canada; Department of Laboratory Medicine and Pathobiology, University of Toronto, Toronto, Ontario, Canada; National Center of Scientific Research ‘Demokritos’, IPC, Athens, 153 10, Greece; Academic Division of Gynecological Oncology, University of Turin, Mauriziano Umberto I° Hospital and Institute for Cancer Research and Treatment (IRCC) of Candiolo, Turin, Italy; Department of Medical Biochemistry, Faculty of Medicine, Mansura University, Egypt

**Keywords:** kallikreins, breast cancer, serine proteases, cancer genes, prognostic factors, predictive markers, *KLK15*, tumour markers, steroid hormones

## Abstract

Many kallikrein genes were found to be differentially expressed in various malignancies, and prostate specific antigen (encoded by the *KLK3* gene) is the best tumour marker for prostate cancer. Prostate specific antigen has recently been shown to be an independent favourable prognostic marker for breast cancer. *KLK15* is newly discovered kallikrein gene that is located adjacent to *KLK3* on chromosome 19q13.4. *KLK15* has 41% similarity to *KLK3* and the encoded protein, hK15, can activate pro-prostate specific antigen. We studied the expression of *KLK15* by real-time quantitative reverse transcriptase–polymerase chain reaction in 202 tissues from patients with breast carcinoma of various stages, grades and histological types. *KLK15* expression was found to be a significant predictor of progression-free survival (hazard ratio of 0.41 and *P*=0.011) and overall survival (hazard ratio of 0.34 and *P*=0.009). When all other known confounders were controlled in the multivariate analysis, *KLK15* retained its prognostic significance. Higher concentrations of *KLK15* mRNA were found more frequently in node negative patients (*P*=0.042). No association was found between *KLK15* expression and any other clinicopathological variable. Further, *KLK15* is an independent prognostic factor of progression-free survival and overall survival in the subgroup of patients with lower grade and those with oestrogen receptor and progesterone receptor negative tumours in both univariate and multivariate analysis. *KLK15* levels of expression were slightly higher (although not statistically significant) in the oestrogen receptor negative and progesterone receptor negative subgroups of patients. *KLK15* is up-regulated by androgens in breast cancer cell lines. Time-course and blocking experiments suggest that this regulation is mediated through the androgen receptor.

*British Journal of Cancer* (2002) **87**, 1294–1300. doi:10.1038/sj.bjc.6600590 www.bjcancer.com

© 2002 Cancer Research UK

## 

The human kallikrein gene family includes 15 serine protease genes, clustered on chromosome 19q13.4. Many kallikreins were found to be differentially expressed in endocrine-related malignancies (Diamandis and Yousef, 2001; Yousef and Diamandis, 2001). KLK10 (also known as the normal epithelial cell-specific 1 gene, NES1) appears to be a novel tumour suppressor, which is down-regulated during breast cancer progression (Goyal *et al*, 1998). KLK6 (zyme/ protease M/neurosin) is overexpressed in primary breast and ovarian cancers (Anisowicz *et al*, 1996) and preliminary studies indicate that it may have utility as a serum biomarker for ovarian carcinoma (Diamandis *et al*, 2000). Two additional kallikrein genes, KLK8 (also known as neuropsin or TADG-14) (Underwood *et al*, 1999; Magklara *et al*, 2001) and the stratum corneum chymotryptic enzyme (Tanimoto *et al*, 1999; Yousef *et al*, 2000c) are up-regulated in ovarian cancer. *KLK12*, *KLK15 and KLK13* seem to be down-regulated in breast cancer (Yousef *et al*, 2000a,b; 2001a).

The gene encoding for prostate specific antigen, *KLK3*, is a member of the human kallikrein family and is located at the centromeric side of the locus. *KLK3* is flanked by the *KLK2* gene (encoding for human glandular kallikrein protein; hK2), and by the *KLK15* (Yousef *et al*, 2001b). In addition to its wide applicability as the best marker for prostate cancer (Diamandis, 1998), hK3, was recently found to be expressed in other tissues, including the female breast (Black and Diamandis, 2000). Prostate specific antigen (PSA) protein in tumour cytosols was found to be an independent marker for favourable prognosis in breast cancer (Yu *et al*, 1995). *KLK15* (encoding for hK15, a protein also named ‘prostinogen’) is the most recently cloned member of the human kallikrein gene family (Takayama *et al*, 2001; Yousef *et al*, 2001b). It is formed of five coding exons and encodes for a serine protease of a predicted molecular weight of about 28 kDa. *KLK15* shares a high degree of structural similarity with *KLK3* and other kallikreins. At the protein level, hK15 has 40% identity and 53% similarity with hK3, which is comparable to the degree of similarity between hK3 and other kallikreins (except hK2). It also has ∼50% similarity with hK9 and hK11. Similarly to hK3, but unlike other trypsin-like serine proteases, hK15 does not have an aspartate residue in the substrate-binding pocket, suggesting a chymotrypsin-like substrate specificity. We have previously shown preliminarily that *KLK15* is up-regulated, at the mRNA level, in prostate cancer (Yousef *et al*, 2001b). A recent report indicated that hK15 can readily activate the precursor of PSA by cleaving an amino terminal peptide bond (Takayama *et al*, 2001).

In this article we report for the first time that *KLK15*, like *KLK3*, is an independent favourable prognostic marker for breast cancer. We further investigated the mechanism that regulates *KLK15* expression in breast cancer cell lines and provide evidence that *KLK15* is up-regulated by androgens through the androgen receptor pathway.

## MATERIALS AND METHODS

### Study population

Included in this study were tumour specimens from 202 consecutive patients undergoing surgical treatment for primary breast carcinoma at the Department of Gynecologic Oncology at the University of Turin, Turin, Italy. All specimens were histologically confirmed. Tumour tissues had been frozen in liquid nitrogen immediately after surgery. This study has been approved by the Institutional Review Board of the University of Turin. The patient ages ranged from 25 to 87 with a median of 57 years. Tumour sizes ranged from 0.1 to 15 cm with a median of 2.2 cm. Follow-up information was available for 190 patients (median follow-up period 78 months), among whom 58 (31%) had relapsed and 48 (25%) died. Local relapses (i.e. ipsilateral breast after breast conserving surgery) or relapses in soft tissues (chest wall after mastectomy or in subclavicular lymphnodes) were diagnosed clinically or radiologically and then always confirmed cytologically/histologically by fine needle aspiration or core biopsy. Visceral metastases (lung, liver) were assessed histologically only when their nature was deemed as equivocal on ultrasound (US), computed tomography (TC) and nuclear magnetic resonance (RMN) evaluation. Bone metastasis were detected clinically or by routine bone scans and then always confirmed by radiograms, TC or RMN. The histological type and steroid hormone receptor status of each tumour as well as the number of positive axillary nodes were established at the time of surgery, as shown in Table 1

**Table 1:**
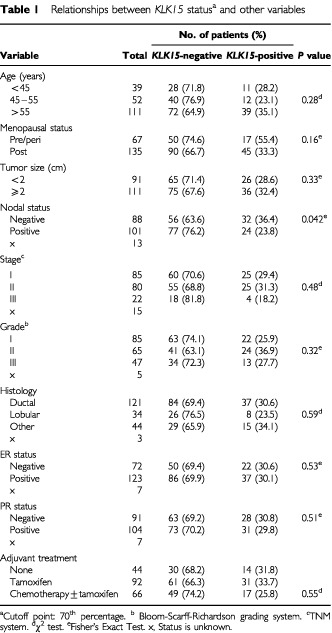
Relationships between *KLK15* status^a^ and other variables

. Out of the 202 patients, 121 (60%) had ductal carcinoma, 34 (17%) lobular carcinoma and 44 (22%) had other histological types (the histological type was not known for three patients). Patients from clinical stages I–III were included in the study, with clinical staging determined according to the TNM classification. Grading of tumours was done according to the Bloom-Scarff-Richardson grading system (Bloom and Richardson, 1957). Forty-four patients (22%) received no adjuvant treatment, 92 (46%) received tamoxifen, and 66 (33%) received chemotherapy with or without tamoxifen. Oestrogen receptor (ER) and progesterone receptor (PR) status was established as described by the European Organization for Research and Treatment of Cancer (EORTC, (1980)).

### Total RNA extraction and cDNA synthesis

Tumour tissues were minced with a scalpel, on dry ice, and transferred immediately to 2 ml polypropylene tubes. They were then homogenised and total RNA was extracted using Trizol™ reagent (Gibco BRL) following the manufacturer's instructions. The concentration and purity of mRNA were determined spectrophotometrically. Two micrograms of total RNA was reverse-transcribed into first strand cDNA using the Superscript™ preamplification system (Gibco BRL). The final volume was 20 μl.

### Quantitative real-time polymerase chain reaction and continuous monitoring of polymerase chain reaction products

Based on the published genomic sequence of *KLK15* (GenBank accession no. AF242195), two gene-specific primers were designed (15-F3: 5′-TGT GGC TTC TCC TCA CTC TC-3′ and 15-R3 5′-AGG CTC GTT GTG GGA CAC-3′). These primers spanned more than two exons to avoid contamination by genomic DNA.

Real-time monitoring of polymerase chain reaction (PCR) reaction was done using the LightCycler™ system (Roche Molecular Systems, Indianapolis, USA) and the SYBR Green I dye, which binds preferentially to double stranded DNA. Fluorescence signals are proportional to the concentration of the product and are measured at the end of each cycle rather than after a fixed number of cycles. The higher the starting quantity of the template, the earlier a significant increase in fluorescence is observed (Bieche *et al*, 1999). The threshold cycle is defined as the fractional cycle number at which fluorescence passes a fixed threshold above baseline. For each sample, the amount of the target and of an endogenous control (β actin, a housekeeping gene) were determined using a calibration curve (see below). The amount of the target molecule was then divided by the amount of the endogenous reference, to obtain a normalised target value.

### Standard curve construction

**Figure 1:**
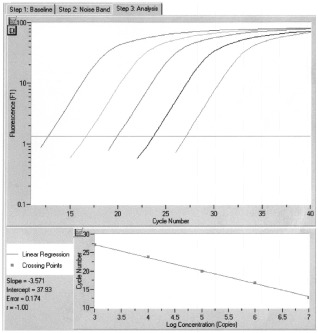
Quantification of *KLK15* gene expression by real-time PCR. (Top) A logarithmic plot of fluorescence signal above the noise level (horizontal line) during amplification. Serial dilutions of a plasmid containing the *KLK15* cDNA were made and an arbitrary copy number was assigned to each sample according to the dilution factor. (Bottom) The crossing points (cycle number), plotted against the log of copy number to obtain a standard curve. For details, see text.

The full-length mRNA sequence of the *KLK15* gene was amplified by PCR using gene-specific primers, and the PCR product was cloned into the TOPO TA cloning vector (Invitrogen, Carlsbad, CA, USA) according to manufacturer's instructions. Plasmid was purified using a Mini-prep kit (Qiagen Inc., Valencia, CA, USA). Different standard curves for actin and *KLK15* were constructed using serial dilutions of the plasmid as described elsewhere (Bieche *et al*, 1999). The standard curve samples were included in each run. The LightCycler software automatically calculates the standard curve by plotting the starting dilution of each standard sample *vs* the threshold cycle, and the sample concentrations were then calculated accordingly (Figure 1

).

### PCR amplification

The PCR reaction was carried out on the LightCycler™ system. For each run, a master mixture was prepared on ice, containing 1 μl of cDNA, 2 μl of LC DNA Master SYBR Green 1 mix, 50 ng of primers and 1.2 μl of 25 mM MgCl_2_. After the reaction mixture was loaded into the glass capillary tube, the cycling conditions were carried out as follows: initial denaturation at 94°C for 10 min, followed by 45 cycles of denaturation at 94°C for 0 s, annealing at 63°C for 5 s, and extension at 72°C for 30 s. The temperature transition rate was set at 20°C s^−1^. Fluorescent product was measured by a single acquisition mode at 88°C after each cycle. A melting curve was then performed by holding the temperature at 70°C for 30 s followed by a gradual increase in temperature to 98°C at a rate of 0.2°C s^−1^, with the signal acquisition mode set at step. To verify the melting curve results, representative samples of the PCR products were sequenced.

### Statistical analysis

Patients were subdivided into groups based on different clinical or pathologic parameters and statistical analyses were performed using SAS software (SAS Institute, Cary, NC, USA). A cutoff point equal to the detection limit (70th percentile) was used. According to this cutoff, *KLK15* expression was classified as positive or negative and associations between *KLK15* status and other qualitative variables were analysed using the chi-square (χ^2^) or the Fisher's Exact Test, where appropriate. The analysis of differences in *KLK15* values between groups of patients was performed with the non-parametric Mann-Whitney *U*-test or Kruskal-Wallis tests. In this analysis, *KLK15* was used as a continuous variable. The cutoff value for tumour size was 2 cm. Lymph node status was either positive (any positive number of nodes) or negative. Age was categorised into three groups: less than 45 years, 45 to 55 years and greater than 55 years. Survival analyses were performed by constructing Kaplan-Meier DFS and OS curves (Kaplan and Meier, 1958) and differences between curves were evaluated by the log-rank test, as well as by estimating the relative risks for relapse and death using the Cox proportional hazards regression model (Cox, 1972). Cox analysis was conducted at both univariate and multivariate levels. Only patients for whom the status of all variables was known were included in the multivariate regression models, which incorporated *KLK15* and all other variables for which the patients were characterised. The multivariate models were adjusted for patient age, nodal status (pathological nodal classification), tumour size, grade, histological type and ER and PR status.

### Breast cancer cell lines and hormonal stimulation experiments

In order to test the hypothesis that *KLK15* is under steroid hormonal regulation, we examined the effect of different steroids on *KLK15* expression in different cell line models. The breast cancer cell lines BT-474, BT-20 and MCF-7 were purchased from the American Type Culture Collection (ATCC, Rockville, MD, USA). Cells were cultured in RPMI media (Gibco BRL, Gaithersburg, MD, USA) supplemented with glutamine (200 mmol l^−1^), foetal bovine serum (10%), in plastic flasks, to near confluency. The cells were then aliquoted into 24-well tissue culture plates and cultured to 50% confluency. Twenty-four hours before the experiments, the culture media were changed into media containing 10% charcoal-stripped foetal bovine serum. For stimulation experiments, various steroid hormones dissolved in 100% ethanol were added into the culture media, at a final concentration of 10^−8^ M. Cells stimulated with 100% ethanol were included as controls. The cells were grown for 24 h, then harvested for mRNA extraction.

### Blocking and kinetic experiments

In order to examine whether *KLK15* regulation occurs directly through the androgen receptor (AR), blocking and kinetic experiments were performed. Blocking experiments were performed as follows: (a) stimulation by the AR blocker alone (two AR blockers were used: RU56,187 and nilutamide), at three different concentrations (10^−6^–10^−8^ M), (b) Stimulation of cells by dihydrotestosterone (DHT) alone at a concentration of 10^−8^ M. These concentrations were chosen based on our previous dose-response studies for *KLK15* in the same cell lines (Zarghami *et al*, 1997; Magklara *et al*, 2000). (c) Addition of the blocker to the cells at three different concentrations, incubation for 1 h and then adding DHT at 10^−8^ M. Ethanol-only stimulated cells were included as controls in order to assess baseline *KLK15* expression. Cells were harvested for analysis after 24 h. For kinetic experiments, the BT-474 cell line was stimulated with DHT at a final concentration of 10^−8^ M and then harvested at 0, 2, 6, 12 and 24 h. Control cells with ethanol alone were included for all time points. All experiments were repeated twice.

## RESULTS

### *KLK15* and breast cancer survival

Out of the 202 patients included in this study, follow-up information was available for 190 patients, among whom 58 (31%) had relapsed and 48 (25%) died. Of 202 breast tumours examined, 62 (30%) were classified as positive for *KLK15* expression. In Table 1 we present the association of *KLK15* status of the tumour (negative or positive) with various clinicopathological variables. We found no statistically significant associations between *KLK15* status and age, menopausal status, tumour size, stage (despite a trend for stage III patients to be less frequently *KLK15*-positive), grade, histological type, ER or PR status and adjuvant chemotherapy. However, node-negative patients were more frequently *KLK15*-positive (*P*=0.042).

The strength of the association between each clinicopathological variable and the progression-free survival (PFS) and overall survival (OS) are shown in the univariate analysis of Table 2

**Table 2:**
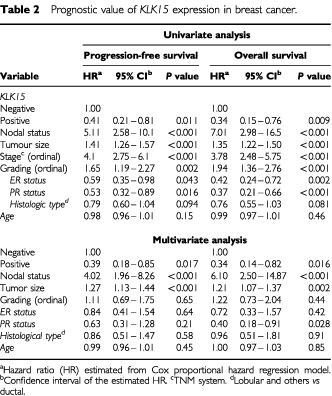
Prognostic value of *KLK15* expression in breast cancer

**Figure 2:**
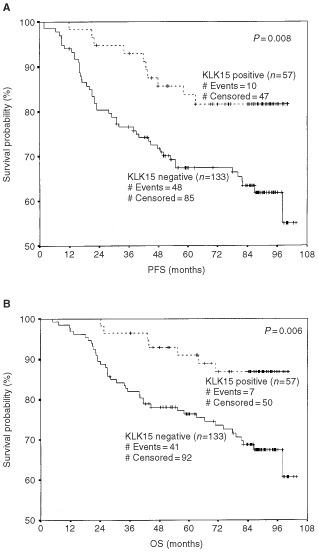
Kaplan-Meier survival curves showing progression-free survival (PFS) (**A**) and Overall survival (OS) (**B**) for patients with *KLK15* positive and *KLK15* negative tumours. For details, see text.

. Nodal status, tumour size, stage and grade of the disease and steroid hormone receptor status were significant predictors for the PFS and OS, as expected. In addition to these known factors, *KLK15* expression was found to be a significant predictor of progression-free survival (hazard ratio of 0.41 and *P*=0.011) and overall survival (hazard ratio of 0.34 and *P*= 0.009). Kaplan-Meier survival curves (Figure 2

) also demonstrate that patients with *KLK15* positive tumours have significantly longer progression-free survival (*P*=0.008) compared to those who are *KLK15* negative. Similar results were found with respect to the overall survival (*P*=0.006) (Figure 2B).

When all parameters were included in the Cox model (multivariate analysis, Table 2), nodal status and tumour size retained their prognostic significance. *KLK15* expression also retained its PFS and OS prognostic significance (hazard ratio of 0.39 and 0.34, and *P* value of 0.017 and 0.016, respectively). A weak association of OS with PR was also seen (*P*=0.028).

Table 3

**Table 3:**
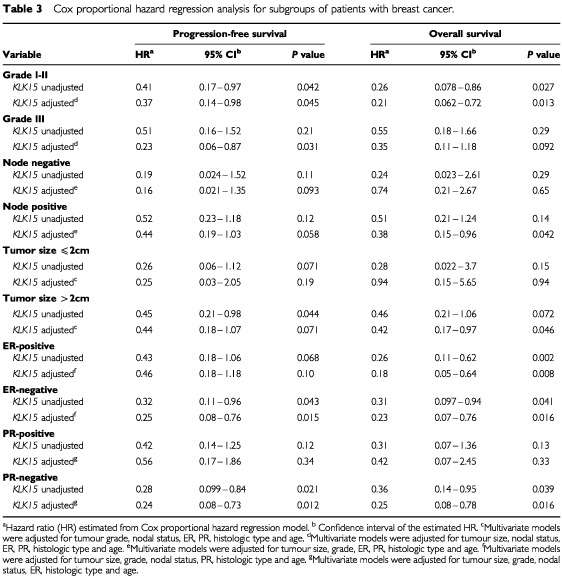
Cox proportional hazard regression analysis for subgroups of patients with breast cancer

shows Cox proportional hazard regression analysis for *KLK15* expression in breast cancer patients stratified by tumour grade, nodal status, tumour size, and ER and PR status. After adjusting for known prognostic confounders, *KLK15* was found to be a significant prognostic factor for both PFS and OS in the subgroup of patients with low grade (grade I–II) and those with ER-negative and PR-negative tumours. Hazard ratios derived from the Cox regression analysis and related to PFS and OS were 0.37 (*P*=0.045) and 0.21 (*P*=0.013), respectively, for those with low-grade tumours, 0.25 (*P*=0.015) and 0.23 (*P*=0.016), respectively, for ER negative tumours, and 0.24 (*P*=0.012) and 0.25 (*P*=0.016), respectively, for PR-negative tumours.

### Hormonal regulation of the *KLK15* gene

In order to explore the mechanism of *KLK15* gene regulation, we identified the promoter of this gene by aligning its genomic sequence against the Human Genome Project sequence database. DNA sequence analysis using different promoter detection algorithms indicated the presence of a putative androgen response element (GGGACAgggGGTCCT) at position −298. This element is comparable to the consensus androgen response element (GG (T/A) ACAnnnTGTTCT). Interestingly, two of the three nucleotides that are different from the consensus ARE (underlined) are also different in the response elements of both PSA and KLK2. We thus hypothesised, as is the case with other kallikreins, that *KLK15* may be up-regulated by androgens through the AR pathway.

**Figure 3:**
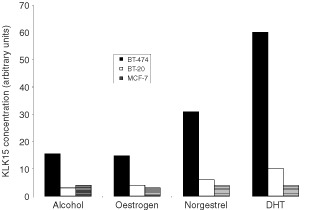
*KLK15* mRNA concentration in three different breast cancer cell lines 24 h after stimulation with steroids at a concentration of 10^−8^ M. DHT, dihydrotestosterone.

**Figure 4:**
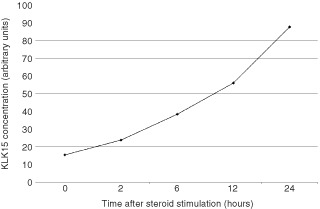
A plot showing the concentration of *KLK15* in the BT-474 breast cancer cell line before (0) and at 2, 6, 12 and 24 h after stimulation with dihydrotestosterone at a concentration of 10^−8^ M.

**Figure 5:**
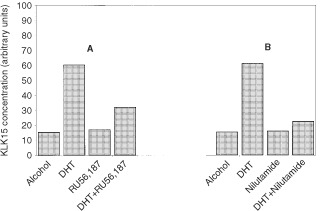
*KLK15* mRNA concentration in the BT-474 cell line 24 h after dihydrotestosterone (DHT) stimulation with and without blocking with (**A**) RU 56,187 and (**B**) nilutamide. Blockers were added at a concentration of 10^−6^ M; DHT was added 1 h later, at a concentration of 10^−8^ M

In order to test this hypothesis, we examined *KLK15* expression in three breast cancer cell lines with variable receptor content. As shown in Figure 4

, *KLK15* was up-regulated by androgens (DHT) and, to a lesser extent, by progestins in the steroid hormone receptor-positive BT-474 cell line. On the other hand, no significant up-regulation of *KLK15* expression was found in the BT-20 cell line, which is known to be devoid of steroid hormone receptors. Also, the MCF-7 cell line did not show any up-regulation of *KLK15* expression with either androgens or progestins (Figure 3

). We have previously confirmed, by Western blot and ligand binding assays, that the cell line has very low androgen receptor content (our data, submitted for publication). Kinetic studies with BT-474 cells showed that *KLK15* up-regulation was detectable as early as 2 h after hormonal stimulation and the level increased gradually over time up to 24 h (Figure 4). These results are similar to those previously reported for *KLK2* and *KLK3* in the same cell lines (Magklara *et al*, 2000). In order to further confirm that this androgen up-regulation occurs through the AR, we conducted blocking experiments, as described in details in Materials and Methods (Figure 5

). Both anti-androgens used (RU 56,187 and nilutamide) were able to block the stimulatory effect of DHT (by about 50–77%). Taken together, these data suggest that *KLK15* is up-regulated by androgens through the AR pathway.

## DISCUSSION

Tumour markers assist in evaluating cancer risk, diagnosis, prognosis or response to treatment (Stearns *et al*, 1998). For breast cancer, the hormone receptor status is the only recommended marker for routine use by the American Society of Clinical Oncology (Smith *et al*, 1999) and the College of American Pathologists Consensus Statement (Fitzgibbons *et al*, 2000). Additional classical prognostic markers, including lymph node status, tumour size and stage, have also proven prognostic importance (Fitzgibbons *et al*, 2000). Many other potential prognostic markers have been identified, including p53, c-erbB2, BCL-2, CEA, CA15.3, CA27.29, cathepsin D and polyadenylate polymerase (Norberg *et al*, 1996; ASCO, (1998); Scorilas *et al*, 1999a,b; 2000; Fitzgibbons *et al*, 2000; Hamilton and Piccart, 2000). Markers may be predictive of treatment response e.g., HER-2 evaluation is useful for selection of patients for Herceptin therapy (Hamilton and Piccart, 2000). In addition, the identification of new prognostic/predictive markers will contribute to more optimal patient sub-grouping and individualisation of treatment strategies (Hamilton and Piccart, 2000). Furthermore, there is now growing interest in neural networks which show promise of combining weak, but independent, information from various biomarkers to produce a prognostic index that is more informative than each individual biomarker alone (Clark *et al*, 1994). In this study, we show that *KLK15* expression has independent and favourable prognostic value in breast cancer. One important consideration that must be taken into account is that large percentage of our study population received adjuvant therapy, which may be a source of bias.

*KLK15* expression is mainly under androgen regulation through the AR, and to a lesser extent by progestin. Breast cancer is a hormone-dependent malignancy (Russo and Russo, 1998), and androgens have been used for treatment of breast cancer, with a therapeutic efficiency comparable to current hormonal therapies such as tamoxifen (Tormey *et al*, 1983). Androgen receptors are present in 70–90% of primary breast tumours (Soreide *et al*, 1992), and AR is the sole steroid hormone receptor expressed in about 25% of metastatic deposits (Lea *et al*, 1989). In addition, medroxyprogesterone acetate (MPA), a synthetic progestin, is a commonly used second line hormonal therapy following failure of tamoxifen. The androgenic side effects of MPA suggest that its action may in part be mediated by the AR. Identification of androgen-regulated genes that mediate the growth-inhibitory effects of androgens may enable more precise prediction of the response to hormonal therapies and define potential new targets for breast cancer treatment (Birrell *et al*, 1998). Such new treatments may be particularly important in metastatic disease, where the AR is often the sole steroid receptor expressed.

The prognostic value of *KLK15* is similar to that of hK3 (PSA) which is also an independent marker of favourable prognosis in breast cancer (Yu *et al*, 1995). In addition to the structural similarity at the mRNA and protein levels, *KLK3*, *KLK2* and *KLK15* have many common features. They are all serine protease genes, located adjacent to each other on chromosome 19q13.4. In addition, all are up-regulated by androgens and progestins in breast cancer cell lines. It is known that hK2 and hK15 can activate hK3 (Takayama *et al*, 1997, 2001). Taken together, we hypothesise that these three kallikreins all regulated by androgens, may play a role in cell proliferation of the breast. In addition, simultaneous measurements of multiple kallikrein levels in tumour tissues may have more prognostic value than that of each individual kallikrein.
